# Sex Differences in Memory: Do Female Reproductive Factors Explain the Differences?

**DOI:** 10.3389/fendo.2022.837852

**Published:** 2022-04-22

**Authors:** Jie Li, Wenting Hao, Chunying Fu, Chengchao Zhou, Dongshan Zhu

**Affiliations:** ^1^ Centre for Health Management and Policy Research, School of Public Health, Cheeloo College of Medicine, Shandong University, Jinan, China; ^2^ NHC Key Laboratory of Health Economics and Policy Research (Shandong University), Jinan, China; ^3^ Department of Epidemiology, School of Public Health, Cheeloo College of Medicine, Shandong University, Jinan, China

**Keywords:** cognitive impairment, cognitive function, estrogen, menarche, menopause

## Abstract

**Background:**

The sex differences in memory impairment were inconclusive, and the effect of female reproductive factors (age at menarche, age at menopause, and reproductive period) on the differences was not clear. We aimed to examine the sex differences in objective and subjective memory impairment in postmenopausal women and age- and education-matched men and explore whether the differences were differed by female reproductive factors.

**Methods:**

Data were obtained from the China Health and Retirement Longitudinal Study. Using the case–control matching method, 3,218 paired postmenopausal women and men matched for age and education were selected. Memory was assessed using the three-word recall task and a self-rated question. Poisson regression models with a robust error variance were used.

**Results:**

The relative risk was 1.22 (95% confidence interval 1.08–1.38) for objective memory impairment in women compared with men (23.87% vs. 27.36%), and 1.51 (1.36–1.67) for subjective memory impairment (39.34% vs. 28.25%) after adjusting the confounders. The higher risk of objective memory impairment in women was different among groups of age at menarche in a linear pattern, with younger age at menarche associated with higher risks of objective memory impairment (*p* < 0.001 for trend). It was also different among groups of menopausal age and reproductive period in an approximate U-shaped pattern, with a similar risk of objective memory with men in women menopause at 52–53 years and having a reproductive period of 31–33 years and higher risks in women with earlier or later menopause (RRs raging form 1.17 to1.41) and a shorter or longer period of reproduction (RR, 1.23–1.29). The higher risks of subjective memory impairment in women were not different among different groups of reproductive factors.

**Conclusions:**

Postmenopausal women were at an increased risk of objective and subjective memory impairment than men. The higher risks in objective memory, but not subjective memory, were varied by age at menarche, age at menopause, and reproductive periods, which may help understand the underlying mechanisms of sex differences in cognitive ageing and guide precise intervention to preventing dementia among older women and men.

## 1 Introduction

With the rapid increase of the older population, cognitive decline is becoming an important topic in relevant areas of clinic and research. Among various domains of cognition, episodic memory impairment was a prominent risk factor of future mild cognitive impairment (1) and dementia (2), and it could increase mortality at 10 years in the older adults without dementia (3). It was also suggested as a required clinical phenotype in the diagnostic criteria for Alzheimer’s disease (4). Episodic memory, which was commonly assessed using word-list tasks measuring verbal episodic memory in clinical and research, was an objective indicator of memory. Meanwhile, self-reported memory, a subjective indicator of memory, has been getting increasing attention recently. It was a key diagnostic criterion for mild cognitive impairment and could be a predictor of future dementia (5, 6). Therefore, both objective and subjective memory were worthy to be considered when studying cognitive decline.

Sex differences have been reported in risk of dementia in older adults (7, 8). Although the risk of dementia was consistently reported to be higher in women than in men (7, 8), the sex differences in performance of verbal episodic memory tests were not clear. Some reported a female advantage in verbal memory among old adults (9–12), while others reported an opposite (13, 14) or no difference (15). Compared with objective memory, subjective memory got much less attention and showed inconsistent findings (16–18). As cognition declines with ageing and is protected by cognitive reserve, when the sex-specific association was compared, age- and education- (a primary proxy of cognitive reserve) matched men and women are necessary to exclude the bias (19, 20). Another noticeable bias was from cognitive reserve, which often used education as the primary proxy. Illustrating sex differences in objective and subjective memory is important, as it was required for a more precise intervention to improve cognition and prevent dementia in female and male older adults, respectively (21).

The factors related to sex differences in memory are however not well understood. Latest evidence suggests that some female-specific factors might contribute to the difference between sexes (22, 23). Compared with men’s gradual loss of sex hormone, women experience a sharp decline in sex hormone (estrogen) during menopause (24). Estrogen has a protective effect on the central nervous system (25, 26), and the estrogen decline during menopause may accelerate the aging of the central nervous system, affecting cognitive function, such as memory (27). Whether estrogen exposure in women moderates the sex-specific association with memory is unclear. As endogenous estrogen exposure is difficult to be assessed directly in population-based studies, reproductive factors (e.g., age at menarche, age at menopause, reproductive period) are usually used as proxy indicators.

Based on the abovementioned, we aimed to examine the sex differences in objective and subjective memory in postmenopausal women and age- and education-matched men and explore the roles of reproductive factors (reproductive period, age at menarche, and age at menopause) between their association.

## 2 Methods

### 2.1 Participants and Data Source

**Figure 1:**
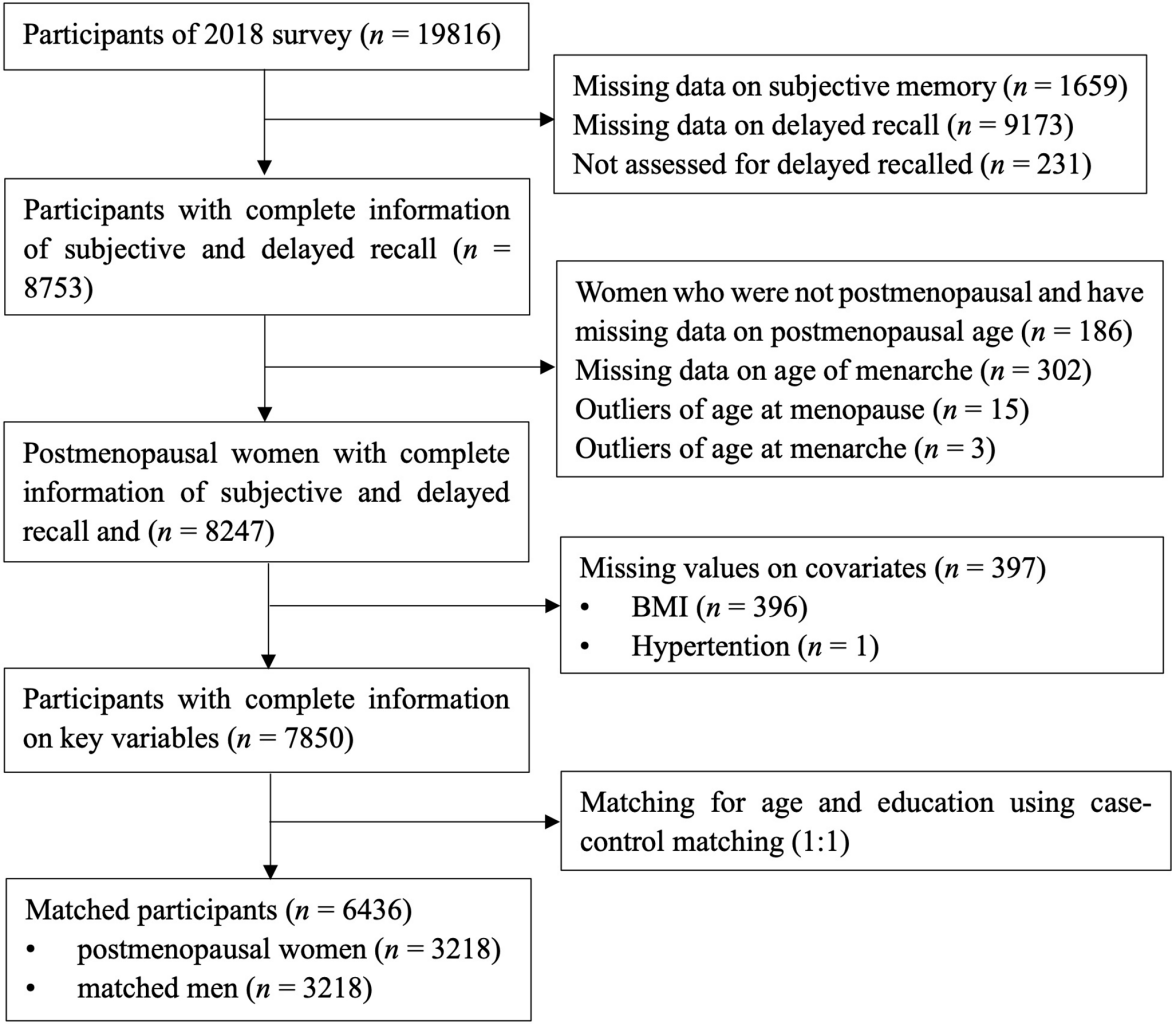
Flow diagram depicting the selection of participants for this study.

Data were extracted from the China Health and Retirement Longitudinal Study (CHARLS). It is a nationally representative longitudinal study, and it collected health and wellbeing information of middled-aged and older adults from 150 counties covering 28 provinces in China. Detailed information about CHARLS has been reported by Zhao et al. (28). The baseline survey was conducted in 2011 and the follow-up survey conducted in 2013, 2015, and 2018. The data of objective and subjective memory from the survey of 2018 were used as the outcome variables in the present cross-sectional study. Information of menarche and menopause was extracted as the exposure variable. The inclusion criteria for the women were postmenopausal before 2018 and having information on memory, menarche, and menopause. There were 7,850 participants (3,707 postmenopausal women and 4,143 men) with complete information on objective and subjective memory, age of menarche, and age of menopause. The matching method (1:1) was used to match women with men on age ± 3 and education, with sets of sampling of no replacement, priority to exact matches, and a randomized case order when drawing matches. A total of 6,436 participants composed of 3,218 postmenopausal women and 3,218 age- and education-matched men were included in the analyses. See **Figure 1** for a flowchart of the participants’ selection process.

### 2.2 Measures

#### 2.2.1 Objective Memory (Verbal Episodic Memory)

The three-word recall task from the Mini-Mental State Examination (MMSE) was used to assess verbal episodic memory, which was used as an indicator of objective memory in the current study (29). The three-word recall task was a brief measure of memory function, which was widely used for screening cognitive impairment (3, 30). In the test, three words “Ball, Flag, and Tree” in Chinese were presented to the participants with an instruction that they would be asked to recall the words after the other parts of MMSE were completed. A cutoff score of less than two out of three words was used to determine memory impairment (31).

#### 2.2.2 Subjective Memory

Subjective memory was assessed using the question, “How would you rate your memory at the present time? Would you say it is excellent, very good, good, fair or poor?” Participants could then rate their memory on a five-point scale from 1 = “excellent” to 5 = “poor.” Participants who rated their memory as poor was defined as subjective memory impairment in the analyses.

#### 2.2.3 Reproductive Factors

Age at menarche and age at menopause were reported by the participants. Age at menarche was defined as menopause as ≥12 consecutive months of amenorrhea. It was categorized into ≤13, 14–15, 16–17, and ≥18 years. Age at menopause was divided into <45, 45–48, 49–51, 52–53, and ≥54 years. The reproductive period was produced by subtracting age at menarche from age at menopause and was categorized by quartiles of the period.

#### 2.2.4 Covariates

Age, education, marital status, area of residence, smoking status, drinking status in the past year, physical activity, body mass index (BMI), and history of hypertension, diabetes, and stroke were used as covariates as they have been shown to be associated with memory (8, 15, 32). Education was dichotomized as < middle school and ≥ middle school. Marital status was dichotomized as not married and married/widowed. Areas of residence included rural and urban areas. Smoking status was divided into never smoking and past/current smoking. Drinking status of the last year was divided into drinking and no drinking. Physical activity was determined by whether doing moderate/vigorous physical activities at least 10 min continuously in a usual week. Height and weight used to compute BMI were measured by the investigators following the standard instruction of the survey. Information on height and weight collected in 2015 was used as it was the most recent measure of height and weight. History of hypertension, diabetes, and stroke was determined by questions of “Have you been diagnosed with hypertension/diabetes or high blood sugar/stroke by a doctor?”

### 2.3 Statistical Analyses

The characteristics of the participants were described using means (standard deviations) and frequencies (percentages) for continuous variables and categorical variables, respectively. Sex differences on sociodemographic variables were tested using independent *t* tests for continuous variables and chi-square tests for categorical variables. Poisson regression models with a robust error variance were used to examine the association of sex, age at menarche, age at menopause, and reproductive period with objective and subjective memory, providing estimates of relative risks (RRs) and 95% confidence intervals (CIs), as the prevalence of memory impairment in this study is more than 10% (33). We first tested the crude model without covariates, then two adjusted models were conducted. First, sociodemographic characteristics, including age, education, marital status, and residence, were adjusted; second, health-related factors (smoking status, drinking status, BMI, physical activities, and history of hypertension, diabetes, and stroke) were adjusted in addition. The association between reproductive factors and memory in women was further examined, controlling all the abovementioned covariates. In those models, male participants were used as the reference group to be compared with groups of women with different ages at menarche, ages at menopause, and reproductive periods. Additionally, age at menarche and age at menopause were mutually adjusted in the analyses among women. Analyses were performed using Stata version 14.0 (Stata Corporation, College Station, TX).

A series of sensitivity analyses were conducted: (i) the association between sex, reproductive factors, and memory was tested in the whole sample of 7,850 participants before matching by age and education; (ii) depression was added as a potential cofounder as it was found to impact cognitive function (8, 32, 34, 35); and (iii) the small proportion of participants who had a history of stroke was excluded as cerebrovascular events, including stroke, might be linked to early menopause and also accelerate the pathology progress of cognitive impairment (36, 37).

## 3 Results

### 3.1 Sample Characteristics

There were 3,218 postmenopausal women and 3,218 men matched for analyses. The sample characteristics are presented in **Table 1**. The mean age at memory evaluation was 68.73 ± 6.37 years of the whole sample, ranging from 50 to 95. There were 80.61% of them having an education lower than middle school, 56.25% doing physical activity, and 9.12% having a history of stroke. There were no significant differences in age, education, physical activity, and history of stroke between women and men. Compared with the proportion in men, women had a lower proportion of being married (women 72.62% vs. men 86.92%), being a past/current smoker (10.72% vs. 82.88%), and drinking in the past year (14.01% vs. 50.75%), but a higher proportion of being from an urban area (28.06% vs. 20.76%) and having a history of hypertension (49.72% vs. 46.67%) and diabetes (20.54% vs. 13.80%).

**Table 1:** Characteristics of the study population (*n* = 6,436).

Variables	All	Women(*n* = 3218)	Men(*n* = 3218)	*t*/*χ^2^*	*p*
Age at memory measurement, years, mean ± SD	68.73 ± 6.37	68.66 ± 6.38	68.80 ± 6.37	-0.87	0.384
Highest level of education, *n* (%)				0.00	1.000
Low education (< middle school)	5,188 (80.61)	2,594 (80.61)	2,594 (80.61)		
High education (≥ middle school)	1,248 (19.39)	624 (19.39)	624 (19.39)		
Marital status, *n* (%)				203.73	<0.001
Married	5,134 (79.77)	2,337 (72.62)	2,797 (86.92)		
Not married/widowed	1,302 (20.23)	881 (27.38)	421 (13.08)		
Residence, *n* (%)				46.50	<0.001
Rural	4,865 (75.59)	2,315 (71.94)	2,550 (79.24)		
Urban	1,571 (24.41)	903 (28.06)	668 (20.76)		
Smoking status, *n* (%)				3,400.00	<0.001
Never	3,424 (53.20)	2,873 (89.28)	551 (17.12)		
Past/current	3,012 (46.80)	345 (10.72)	2,667 (82.88)		
Drinking in past year, *n* (%)				991.44	<0.001
No	4,352 (67.62)	2,767 (85.99)	1,585 (49.25)		
Yes	2,084 (32.38)	451 (14.01)	1,633 (50.75)		
Physical activity				0.31	0.580
No	2,816 (43.75)	1,397 (43.41)	1,419 (44.10)		
Yes	3,620 (56.25)	1,821 (56.59)	1,799 (55.90)		
Body mass index, kg/m^2^	23.65 ± 3.68	24.24 ± 3.81	23.07 ± 3.45	12.86	<0.001
History of hypertension				5.98	0.014
No	3,334 (51.80)	1,618 (50.28)	1,716 (53.33)		
Yes	3,102 (48.20)	1,600 (49.72)	1,502 (46.67)		
History of diabetes				51.45	<0.001
No	5,331 (82.83)	2,557 (79.46)	2,774 (86.20)		
Yes	1,105 (17.17)	661 (20.54)	444 (13.80)		
History of stroke				0.02	0.897
No	5,849 (90.88)	2,926 (90.93)	2,923 (90.83)		
Yes	587 (9.12)	292 (9.07)	295 (9.17)		

### 3.2 Sex, Reproductive Factors, and Memory

#### 3.2.1 Objective Memory

**Figure 2:**
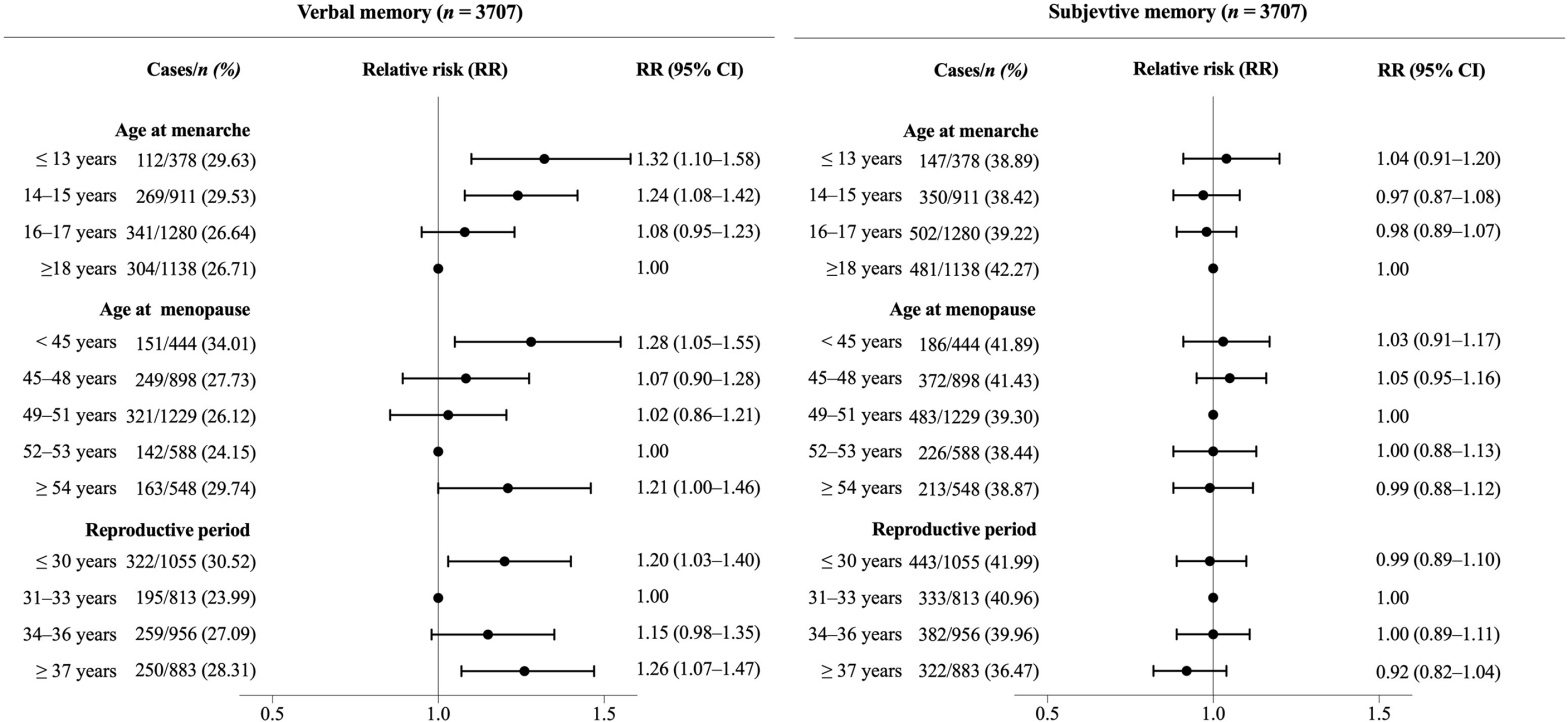
Association between reproductive period, age at menopause, age at menarche, and risk of memory impairment in postmenopausal women. Poisson regression models with a robust error variance were used to estimate relative risks (RRs) and 95% confidence intervals (CIs). All RRs were adjusted for age at memory evaluating, education, marital status, area of residence, smoking status, drinking status in the past year, physical activity, BMI, and history of hypertension, diabetes, and stroke. Age at menarche and age at menopause were mutually adjusted in the analyses.

The incidences of objective memory impairment for women and men were 27.36% and 23.87%, respectively. Women had a 22% higher risk of objective memory impairment than men (RR 1.22, 95% CI 1.08–1.38). The elevated risk was moderated by women’s timing of menarche, menopausal age, and reproductive period. An inverse linear relationship was found between age of menarche and objective memory impairment. Younger age at menarche was associated with higher risks of objective memory impairment (*p* < 0.001 for trend). An approximate U-shaped relationship was observed between age at menopause, length of reproductive period, and objective memory impairment in women. Women who reached menopause at 52–53 years and had a reproductive period of 31–33 years did not differ in risks of objective memory impairment with men, and the risks were higher in women with early (<45 years, 1.41, 1.18–1.68) and late menopause (≥54 years, 1.35, 1.13–1.61) and with reproductive period ≤30 (1.28, 1.10–1.48) and ≥37 years (1.29, 1.10–1.51) (**Table 2**). When only women were included in the analyses, and ages of menarche, ages of menopause, and reproductive period were adjusted for each other, we found similar trends between menarchal or menopausal ages and risk of objective memory. However, the size of estimates (i.e., RR value) was attenuated slightly (**Figure 2**).

**Table 2:** Association between sex, age at menarche, age at menopause, reproductive period, and objective memory impairment (*n* = 6,436).

	Case/*n* (%)	Crude RR (95% CI)	Adjusted RR (95% CI) *^a^*	Adjusted RR (95% CI) *^b^*
Sex				
Women	889/3,218 (27.36)	1.16 (1.07–1.26)	1.16 (1.07–1.26)	1.22 (1.08–1.38)
Men	768/3,218 (23.87)	1.00	1.00	1.00
Age at menarche, years				
≤13	95/327 (29.05)	1.22 (1.02–1.46)	1.33 (1.12–1.59)	1.42 (1.16–1.74)
14–15	229/784 (29.21)	1.22 (1.08–1.39)	1.26 (1.12–1.43)	1.33 (1.14–1.56)
16–17	294/1,121 (26.23)	1.10 (0.98–1.23)	1.11 (0.99–1.25)	1.18 (1.02–1.36)
≥18	271/986 (27.48)	1.15 (1.02–1.30)	1.09 (0.97–1.23)	1.14 (0.98–1.33)
Men	768/3,218 (23.87)	1.00	1.00	1.00
Age at menopause, years				
<45	134/394 (34.01)	1.43 (1.23–1.66)	1.34 (1.15–1.56)	1.41 (1.18–1.68)
45–48	213/777 (27.41)	1.15 (1.01–1.31)	1.14 (1.00–1.30)	1.19 (1.02–1.39)
49–51	283/1,064 (26.60)	1.11 (0.99–1.25)	1.12 (1.00–1.26)	1.17 (1.01–1.36)
52–53	114/498 (22.89)	0.96 (0.81–1.14)	1.02 (0.86–1.21)	1.08 (0.89–1.31)
≥54	145/485 (29.90)	1.25 (1.08–1.45)	1.28 (1.10–1.48)	1.35 (1.13–1.61)
Men	768/3,218 (23.87)	1.00	1.00	1.00
Reproductive period, years				
≤30	284/923 (30.77)	1.29 (1.15–1.45)	1.22 (1.09–1.37)	1.28 (1.10–1.48)
31–33	170/706 (24.08)	1.01 (0.87–1.17)	1.02 (0.88–1.18)	1.06 (0.89–1.26)
34–36	224/819 (27.35)	1.15 (1.01–1.30)	1.17 (1.03–1.33)	1.23 (1.05–1.44)
≥37	211/770 (27.40)	1.15 (1.01–1.31)	1.22 (1.07–1.38)	1.29 (1.10–1.51)
Men	768/3,218 (23.87)	1.00	1.00	1.00

RR, relative risk; CI, confidence intervals.

^a^Adjusted for age, education, marital status, and residence.

^b^Adjusted for smoking status, drinking status, BMI, physical activities, and history of hypertension, diabetes, and stroke, in addition.

#### 3.2.2 Subjective Memory

The rates of subjective memory impairment for women and men were 39.34% and 28.25%, respectively. Women were 1.51 times as men to report poor subjective memory after adjusting the covariates (1.51, 1.36–1.67). The risks of subjective memory impairment across different levels of women’s reproductive factors were 42% to 64% higher than those of men (**Table 3**). Among women with different levels of reproductive factors, women menarche at the age of ≤13 years had the highest risks (1.64, 1.39–1.93) and women with reproductive period ≥37 years had the lowest risks (1.42, 1.25–1.62) compared with men. When ages of menarche, ages of menopause, and reproductive period were adjusted for each other, although similar trends were observed, the sizes of RRs were attenuated to be insignificant (**Figure 2**). The results from the male subjects by using one of the groups of women as reference are shown in **Supplementary Figure 1** to further clarify the comparisons of objective and subjective memory impairment between women of different ages of menarche, ages of menopause, and reproductive years and men.

**Table 3:** Association between sex, age at menarche, age at menopause, reproductive period, and subjective memory impairment (*n* = 6,436).

	Case/*n* (%)	Crude model	Adjusted *^a^* model	Adjusted *^b^* model
Sex				
Women	1,266/3,218 (39.34)	1.39 (1.30–1.49)	1.43 (1.33–1.53)	1.51 (1.36–1.67)
Men	909/3,218 (28.25)	1.00	1.00	1.00
Age at menarche, years				
≤13	128/327 (39.14)	1.39 (1.20–1.60)	1.54 (1.33–1.78)	1.64 (1.39–1.93)
14–15	292/784 (37.24)	1.32 (1.19–1.47)	1.39 (1.25–1.54)	1.47 (1.29–1.67)
16–17	437/1,121 (38.98)	1.38 (1.26–1.51)	1.42 (1.30–1.56)	1.50 (1.34–1.69)
≥18	409/986 (41.48)	1.47 (1.34–1.61)	1.43 (1.31–1.57)	1.50 (1.33–1.70)
Men	909/3,218 (28.25)	1.00	1.00	1.00
Age at menopause, years				
<45	165/394 (41.88)	1.48 (1.30–1.69)	1.46 (1.29–1.66)	1.55 (1.34–1.79)
45–48	318/777 (40.93)	1.45 (1.31–1.60)	1.47 (1.33–1.62)	1.55 (1.37–1.76)
49–51	409/1,064 (38.44)	1.36 (1.24–1.49)	1.40 (1.27–1.53)	1.47 (1.31–1.66)
52–53	193/498 (38.76)	1.37 (1.21–1.55)	1.45 (1.28–1.64)	1.52 (1.32–1.76)
≥54	181/485 (37.32)	1.32 (1.16–1.50)	1.38 (1.21–1.56)	1.44 (1.25–1.67)
Men	909/3,218 (28.25)	1.00	1.00	1.00
Reproductive period, years				
≤30	384/923 (41.60)	1.47 (1.34–1.62)	1.44 (1.31–1.58)	1.53 (1.35–1.72)
31–33	285/706 (40.37)	1.43 (1.29–1.59)	1.46 (1.32–1.62)	1.54 (1.35–1.75)
34–36	322/819 (39.32)	1.39 (1.26–1.54)	1.44 (1.30–1.60)	1.53 (1.35–1.73)
≥37	275/770 (35.71)	1.26 (1.13–1.41)	1.35 (1.22–1.51)	1.42 (1.25–1.62)
Men	909/3,218 (28.25)	1.00	1.00	1.00

RR, relative risk; CI, confidence intervals.

^a^Adjusted for age, education, marital status, and residence.

^b^Adjusted for smoking status, drinking status, BMI, history of hypertension, diabetes, cardiovascular diseases at baseline, physical activities, in addition.

### 3.3 Sensitivity Analyses

Results were similar when the analyses were conducted in the whole unmatched sample of 7,850 participants (**Supplementary Table 1**). When controlling for depressive symptoms in addition, the associations were slightly attenuated and the associations between reproductive years of 34 and 36 and menarche at ages 16–17 and objective memory impairment were no longer statistically significant; however, similar patterns of the associations were found (**Supplementary Table 2**). When participants who had a history of stroke were excluded, the results remained (**Supplementary Table 3**).

## 4 Discussion

This study examined the sex differences in objective and subjective memory impairment and explored the association between reproductive factors and the differences in postmenopausal women and age- and education-matched men using population-based data. There were 27.36% postmenopausal women and 23.87% matched men who experienced objective memory impairment, and 39.34% women and 28.25% men who reported subjective memory impairment. We found that postmenopausal women had higher risks of both objective and subjective memory impairment than men of similar age and education, and the extent of elevated risk was different among different levels of reproductive factors. The findings suggest moderating roles of female reproductive factors in determining the differences in memory function in postmenopausal women and their age- and education-matched men.

We found higher risks of objective and subjective memory impairment in postmenopausal women than men of similar age and education. Lots of previous studies have found women’s performance advantages in verbal memory (9, 12, 38), which were contrary to our findings. The possible reason may be that the sample of this study was Chinese population with older age. They were likely to have the least women advantages in verbal memory because women’s advantages in verbal memory tasks were found to be fewer in populations from Asia than from other countries (e.g., Europe, Oceania, and America), and in older populations than young populations (38). Differences in subjective memory were rarely studied in older adults. A meta-analysis found that women were more sensitive to memory changes than men (13), which may make women more likely to self-rate memory impairment than men. Martinez et al. (39) found that young women reported greater subjective memory impairment than young men. The current results additionally support it in middle-aged and older adults.

The higher risks of objective memory impairment in postmenopausal women than in men were different across age at menarche, age at menopause, and reproductive period. It supports the effect of female reproductive factors on cognitive function (22). Women with a reproductive period of 31–33 years were at the lowest point of risk of objective memory impairment, which were similar to the risk of men, and women with shorter/longer reproductive periods than 31–33 years had higher risks than men. The association was in a U-shaped pattern among women. However, the U-shaped association was not found in previous similar studies (40, 41). One of the possible reasons may be that overall cognitive function was measured in those studies, while we focused on memory. The association between estrogen and cognitive function was found to be different among different cognitive domains, and verbal memory was more likely to be affected (42). They found a trend that a longer duration of reproductive period was associated with a lower risk of cognitive impairment, which was contrary to our results that a longer duration was related with a higher risk of memory impairment among women with a reproductive period longer than 30 years (accounting for 71% of our study population). However, a similar association with our finding was found, showing that a longer reproductive period was related with a higher risk of all-cause dementia and Alzheimer’s disease in a 44-year longitudinal population study (43). It may indicate that memory impairment was a strong predictor of future dementia (1–4).

The risk of objective memory impairment in women who experienced menopause at the age of 52–53 was the lowest one among groups of menopausal age and comparable with the risk in men. Women who reached menopause younger than 52 years or older than 53 years were at a higher risk of objective memory impairment than men. A recent study in Singapore Chinese women found a similar U-shaped association between age at menopause and cognitive impairment with the association in our findings (40). Findings from British women indicated that later age of menopause was linearly associated with better verbal memory (44). Our results of the U-shaped association, in line with the Singapore findings, may help clarify the association between reproductive period and cognitive function.

A novel result was found showing that earlier age at menarche showed an increased risk of verbal memory. It was rarely studied, and a few related studies were either on overall cognitive impairment or dementia, rather than verbal memory. They did not find a significant association with cognitive impairment (40) and dementia (43) or find an inverse association with cognitive impairment (45, 46) with the association in our results. However, findings from Parkinson’s disease (32) and preclinical biomarkers of Alzheimer’s disease (47) supported our results. In the first study, earlier age at menarche was found to be associated with higher risk of Parkinson’s disease, which was also a neurodegenerative disorder, and could resulted in dementia. In the second study, earlier menarche was associated with higher levels of hyperphosphorylated tau and a lower ratio of amyloid-β 1-42/40 in cerebrospinal fluid, which were preclinical markers of Alzheimer’s disease and associated with verbal memory (48).

Women were all at a higher risk of subjective memory impairment than men, while the differences between different groups of age at menarche, age at menopause, and reproductive period were not significant in women. Subjective rated cognitive function was evidenced to be affected by depression (49). Thus, depression was controlled in the sensitivity analyses, and the higher risk remained just with a small decrease. It was consistent with the finding of Martinez et al. (39) that women reported a greater subjective cognitive decline than men. However, Sundermann et al. (17) found no differences between women and men in self-report memory function. It should be noted that men were older than women in their study, which may decrease the men’s self-rated memory. The association between reproductive factors and subjective memory was unclear as there were few studies on it. A finding that women taking estrogen-decreasing treatment had increased cognitive complaints supported a possible association between reproductive factors and subjective cognition (50). More evidence was needed to clarify the association in reproductive factors.

### 4.1 Strengths

A growing literature has indicated that women’s cognitive function was associated with reproductive factors, including age at menarche, age at menopause, and reproductive period (25, 40, 43, 44, 51). However, it focused less on memory, which was a key domain of cognitive ageing. Furthermore, memory declines with increased age and less education, which is the most well-known cognitive reserve factor that affects cognitive function. We thus explored the sex-specific association with memory in age- and education-matched men and women, separating the effect of sex from age and education. Other strengths of the study include the 1:1 matched case–control design of the study, the comprehensive measurement of memory from objective and subjective aspects, and the large number of evaluated individuals based on a national population.

### 4.2 Limitation

Our study also has several limitations. First, there may be residual confounding although age and education were matched and a series of covariates were adjusted, for example, the apolipoprotein E type 4 allele. Second, other reproductive factors affecting endogenous estrogen exposure were not included in the analyses, for example, times of parity and duration of breast feeding. However, the primary ones were analyzed in this study. Further research considering more reproductive factors were needed to validate the current results. Third, there might be some recall biases of age at menarche and age at menopause, especially age at menarche, which happened decades before the survey. The actual levels of estrogen can be used in addition to these reproductive factors in future studies. Fourth, subjective memory was measured using a question that asked the participants to rate their total subjective memory, although this may cover the characteristics of the subjective memory; a more specific and robust measurement was needed in further studies.

## 5 Conclusions

Postmenopausal women were at an increased risk of objective and subjective memory impairment than their age- and education-matched men. The higher risk in objective memory, but not subjective memory, was varied by age at menopause and reproductive periods in a non-liner manner and varied by age at menarche in a linear manner. It supports and further clarifies the association between endogenous estrogen exposure and cognitive function. The results may help identify older women at higher risk of cognitive impairment and understand the underlying mechanisms of sex differences in cognitive decline.

## Data Availability Statement

Publicly available datasets were analyzed in this study. These data can be found here: https://charls.charlsdata.com/pages/data/111/en.html.

## Ethics Statement

The studies involving human participants were reviewed and approved by the Biomedical Ethics Review Committee of Peking University. The patients/participants provided their written informed consent to participate in this study.

## Author Contributions

JL: formal analysis, methodology, writing—original draft. WH: formal analysis, writing—review and editing. CF: formal analysis. CZ: conceptualization, supervision, funding acquisition. DZ: conceptualization, supervision, writing—review and editing. All authors contributed to the article and approved the submitted version.

## Funding

This work was supported by the National Natural Science Foundation of China [Grant number 71774104].

## Conflict of Interest

The authors declare that the research was conducted in the absence of any commercial or financial relationships that could be construed as a potential conflict of interest.

## Publisher’s Note

All claims expressed in this article are solely those of the authors and do not necessarily represent those of their affiliated organizations, or those of the publisher, the editors and the reviewers. Any product that may be evaluated in this article, or claim that may be made by its manufacturer, is not guaranteed or endorsed by the publisher.

## Supplementary Material

Supplementary Figure 1 Comparisons of objective and subjective memory impairment between women of different ages of menarche, ages of menopause, and reproductive years and men by using one of the groups of women as reference.
