# Supplementary material for: Sex Differences in Memory: Do Female Reproductive Factors Explain the Differences?
Source: Front Endocrinol (Lausanne). 2022 Apr 22;13:837852. doi: 10.3389/fendo.2022.837852 (PMC9073013; doi:10.3389/fendo.2022.837852)
Supplement: Supplementary file 1 [file Table_1.pdf]

Supplementary Table 1. Association between sex, age at menarche, age at menopause, reproductive period and memory impairment in the unmatched sample (n = 7850)

	Objective memory		Subjective memory	
	Case/n (%)	Adjusted RR (95% CI)[a]	Case/n (%)	Adjusted RR (95% CI)[a]
Sex				
Women	1026/3707 (27.68)	1.20 (1.07–1.35)	1480/3707 (39.92)	1.51 (1.38–1.67)
Men	917/4143 (22.13)	1.00	1093/4143 (26.38)	1.00
Age at menarche, years				
≤ 13	112/378 (29.63)	1.41 (1.14–1.70)	147/378 (38.89)	1.60 (1.38–1.87)
14–15	269/911 (29.53)	1.32 (1.21–1.52)	350/911 (38.42)	1.49 (1.32–1.68)
16–17	341/1280 (26.64)	1.17 (1.02–1.34)	502/1280 (39.22)	1.49 (1.34–1.67)
≥ 18	304/1138 (26.71)	1.09 (0.95–1.26)	481/1138 (42.27)	1.53 (1.37–1.71)
Men	917/4143 (22.13)	1.00	1093/4143 (26.38)	1.00
Age at menopause, years				
< 45	151/444 (34.01)	1.39 (1.17–1.63)	186/444 (41.89)	1.53 (1.34–1.76)
45–48	249/898 (27.73)	1.19 (1.02–1.37)	372/898 (41.43)	1.56 (1.39–1.76)
49–51	321/1229 (26.12)	1.13 (0.98–1.30)	483/1229 (39.30)	1.50 (1.34–1.67)
52–53	142/588 (24.15)	1.11 (0.93–1.32)	226/588 (38.44)	1.50 (1.31–1.71)
≥ 54	163/548 (29.74)	1.31 (1.11–1.54)	213/548 (38.87)	1.48 (1.29–1.69)
Men	917/4143 (22.13)	1.00	1093/4143 (26.38)	1.00
Reproductive period, years				
≤ 30	322/1055 (30.52)	1.25 (1.09–1.44)	443/1055 (41.99)	1.53 (1.37–1.71)
31–33	195/813 (23.99)	1.04 (0.89–1.22)	333/813 (40.96)	1.55 (1.37–1.74)
34–36	259/956 (27.09)	1.20 (1.04–1.38)	382/956 (39.96)	1.54 (1.38–1.73)
≥ 37	250/883 (28.31)	1.29 (1.12–1.50)	322/883 (36.47)	1.43 (1.26–1.61)
Men	917/4143 (22.13)	1.00	1093/4143 (26.38)	1.00

[a]*Adjusted for age, education, marital status, residence, smoking status, drinking status, BMI, physical activities, and history of hypertension, diabetes, and stroke.*
