# Supplementary material for: Sex Differences in Memory: Do Female Reproductive Factors Explain the Differences?
Source: Front Endocrinol (Lausanne). 2022 Apr 22;13:837852. doi: 10.3389/fendo.2022.837852 (PMC9073013; doi:10.3389/fendo.2022.837852)
Supplement: Supplementary file 2 [file Table_2.pdf]

Supplementary Table 2. Association between sex, age at menarche, age at menopause, reproductive period and memory impairment after additionally controlling for depression (*n* = 6864)

	Objective memory RR (95% CI)	Subjective memory RR (95% CI)
Sex		
Women	1.17 (1.03–1.33)	1.41 (1.27–1.56)
Men	1.00	1.00
Age at menarche, years		
≤ 13	1.39 (1.13–1.70)	1.38 (1.16–1.64)
14–15	1.33 (1.13–1.56)	1.43 (1.25–1.63)
16–17	1.10 (0.94–1.29)	1.39 (1.23–1.57)
≥ 18	1.07 (0.91–1.25)	1.42 (1.26–1.61)
Men	1.00	1.00
Age at menopause, years		
< 45	1.45 (1.21–1.74)	1.37 (1.17–1.60)
45–48	1.18 (1.00–1.39)	1.48 (1.30–1.68)
49–51	1.05 (0.90–1.23)	1.38 (1.22–1.56)
52–53	1.04 (0.86–1.27)	1.39 (1.20–1.61)
≥ 54	1.29 (1.07–1.55)	1.42 (1.22–1.64)
Men	1.00	1.00
Reproductive period, years		
≤ 30	1.25 (1.07–1.46)	1.41 (1.25–1.60)
31–33	1.02 (0.85–1.22)	1.42 (1.25–1.62)
34–36	1.10 (0.93–1.30)	1.48 (1.31–1.68)
≥ 37	1.28 (1.09–1.51)	1.31 (1.14–1.50)
Men	1.00	1.00

6864 out of 7850 participants had valid information on depression.
