# Supplementary material for: Sex Differences in Memory: Do Female Reproductive Factors Explain the Differences?
Source: Front Endocrinol (Lausanne). 2022 Apr 22;13:837852. doi: 10.3389/fendo.2022.837852 (PMC9073013; doi:10.3389/fendo.2022.837852)
Supplement: Supplementary file 3 [file Table_3.pdf]

Supplementary Table 3. Association between sex, age at menarche, age at menopause, reproductive period and memory impairment after excluding participants who had a history of stroke (*n* = 7133)

	Objective memory RR (95% CI)	Subjective memory RR (95% CI)
Sex		
Women	1.20 (1.06–1.36)	1.58 (1.43–1.76)
Men	1.00	1.00
Age at menarche, years		
≤ 13	1.40 (1.15–1.70)	1.69 (1.44–1.98)
14–15	1.31 (1.12–1.52)	1.56 (1.37–1.77)
16–17	1.18 (1.02–1.36)	1.57 (1.39–1.76)
≥ 18	1.10 (0.95–1.28)	1.60 (1.42–1.80)
Men	1.00	1.00
Age at menopause, years		
< 45	1.38 (1.16–1.65)	1.60 (1.38–1.85)
45–48	1.19 (1.02–1.39)	1.63 (1.44–1.85)
49–51	1.13 (0.97–1.31)	1.58 (1.40–1.77)
52–53	1.11 (0.92–1.33)	1.54 (1.34–1.78)
≥ 54	1.31 (1.10–1.57)	1.55 (1.34–1.79)
Men	1.00	1.00
Reproductive period, years		
≤ 30	1.27 (1.10–1.46)	1.61 (1.42–1.81)
31–33	1.03 (0.87–1.22)	1.61 (1.42–1.83)
34–36	1.17 (1.00–1.37)	1.59 (1.40–1.80)
≥ 37	1.31 (1.12–1.53)	1.52 (1.33–1.73)
Men	1.00	1.00
