# Supplementary figures and images for: Sex Differences in Memory: Do Female Reproductive Factors Explain the Differences?
Source: Front Endocrinol (Lausanne). 2022 Apr 22;13:837852. doi: 10.3389/fendo.2022.837852 (PMC9073013; doi:10.3389/fendo.2022.837852)

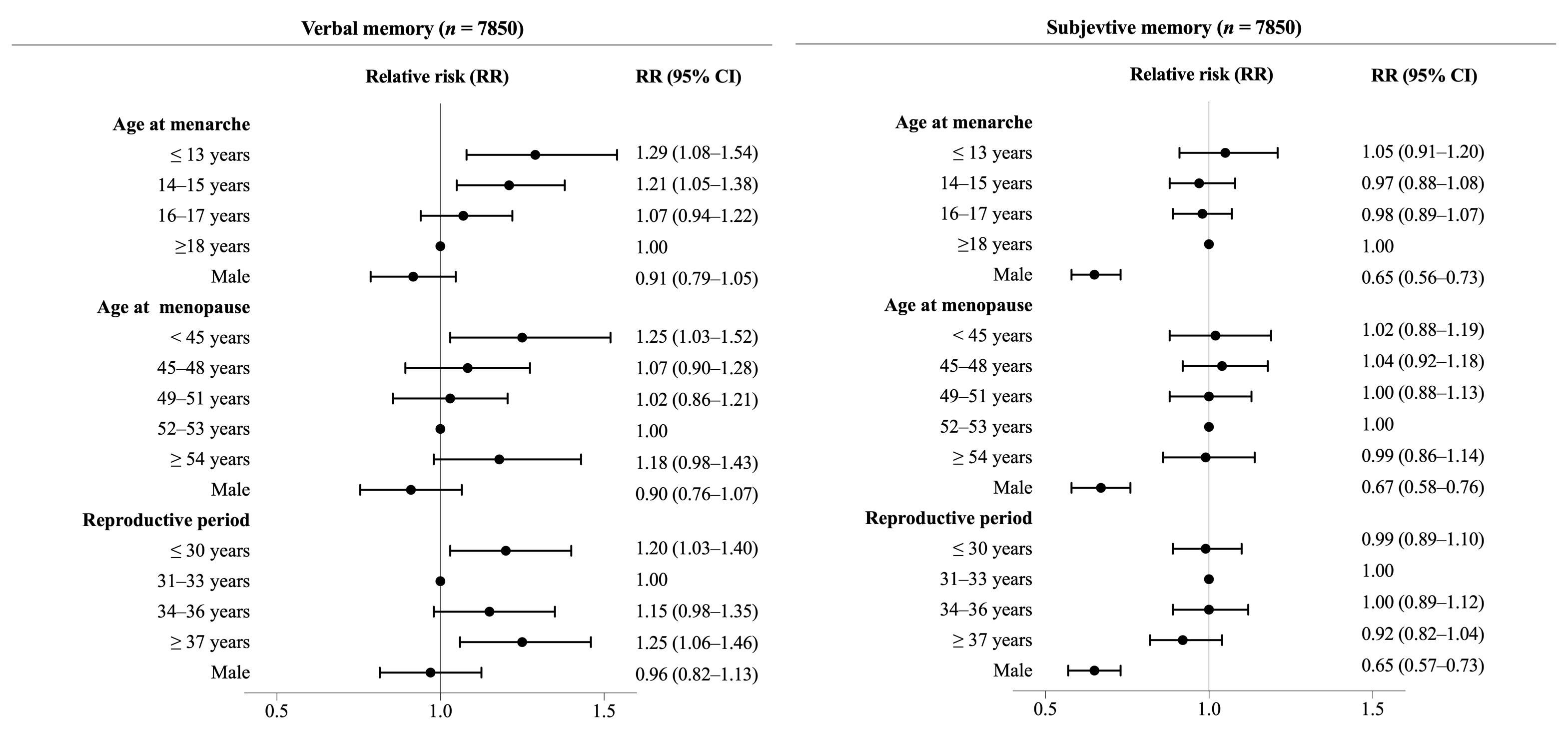

Supplement: Supplementary Figure 1 — Comparisons of objective and subjective memory impairment between women of different ages of menarche, ages of menopause, and reproductive years and men by using one of the groups of women as reference. [file Image_1.tiff]
